# How new quality productivity drives china’s sustainable development: Evidence from dynamic indicator evolution

**DOI:** 10.1371/journal.pone.0338804

**Published:** 2025-12-12

**Authors:** Minhua Lu

**Affiliations:** School of International Relations and Public Affairs, Shanghai International Studies University, Shanghai, China; National Cheng Kung University, TAIWAN

## Abstract

New Quality Productivity (NPQ) plays a pivotal role in driving China’s transition toward sustainable economic development. This study constructs a multidimensional evaluation framework incorporating thirteen indicators across digitalization, green development, and institutional innovation, and utilizes entropy-based analysis of China’s time-series data from 2000 to 2022. The results reveal that NPQ markedly enhances economic efficiency, social inclusiveness, and environmental sustainability through technological progress, optimization of resource allocation, and institutional improvement. The composite index rose sharply from 0.0598 in 2000 to 0.9627 in 2022, representing an average annual growth rate of 12.4%. Weight analyses identify low-carbon technology innovation, digital accessibility, and environmental policy strictness as core drivers, with significant structural advances corresponding to major policy initiatives in 2012, 2016, and 2020. Robustness and statistical tests confirm the reliability of the model and the progressive strengthening of digitalization and green development dimensions over time. This study provides empirical evidence to guide NPQ-related policy formulation and contributes new theoretical perspectives to global sustainable development discourse by highlighting the importance of coordinated technological, environmental, and institutional advancement.

## Introduction

In the face of mounting global challenges, including environmental degradation, resource limitations, and widening social inequalities, the pursuit of sustainable and high-quality economic development has become a central concern for scholars and policymakers. Traditional, resource-intensive growth models often result in ecological harm and unequal distribution of benefits, highlighting the need for productivity paradigms that balance economic efficiency, social equity, and environmental responsibility. Within this context, the concept of New Quality Productivity (NPQ) has attracted increasing attention, particularly in China’s ongoing economic transformation [1].

In this study, NPQ is defined as an advanced, innovation-driven form of productivity resulting from the dynamic integration of digitalization, green development, and institutional innovation. In contrast to traditional productivity, which relies predominantly on capital, labor, and raw resources, NPQ is characterized by the adoption of cutting-edge technologies, environmentally sustainable practices, and adaptive governance frameworks [2]. These three dimensions not only represent distinct channels of productive upgrading but also interact synergistically to reshape production, distribution, and governance systems. Although similar concepts have been addressed in research on endogenous growth, green innovation systems, and institutional economics, most existing studies examine these drivers in isolation or through static, cross-sectional analyses, leaving a gap in understanding the co-evolution and synergistic effects of NPQ’s multi-dimensional factors on sustainable development over time [3–5].

To address this gap, a comprehensive analytical framework is proposed to evaluate the evolution and impact of NPQ from an integrated, longitudinal perspective. Drawing upon a novel dataset covering key phases of China’s transformation from 2000 to 2022, a multi-dimensional indicator system is constructed, including digital infrastructure, eco-innovation, and institutional quality, while an entropy-based methodology assesses indicator weights and evolutionary dynamics. This approach surpasses single-dimensional or purely subjective assessments, enabling a more objective comparison of the relative importance and interplay of digital, green, and institutional factors across various policy stages.

Furthermore, the analysis situates China’s experience within a global context by benchmarking it against established frameworks, such as OECD productivity indices and the United Nations Sustainable Development Goals (SDGs). This comparative perspective highlights both the distinctiveness and potential international applicability of the NPQ model, providing theoretical and policy insights relevant for a broad range of developing and emerging economies.

In summary, this study makes three major contributions. It offers a clear, academically-grounded definition and operationalization of NPQ, emphasizing the mutual reinforcement of digitalization, green development, and institutional innovation. It employs a dynamic, entropy-based framework to identify the temporal evolution and driving mechanisms of NPQ in relation to sustainable development. Finally, it provides evidence-based recommendations for high-quality development policy, contributing to the advancement of global sustainability objectives.

## Literature review

The concept of New Quality Productivity (NPQ) originated in China’s recent modernization and economic transformation discourse, and has rapidly attracted academic attention due to its potential to address the limitations of resource-intensive growth models [6,7]. NPQ is defined as a multi-dimensional productivity paradigm emphasizing technological innovation, digital transformation, green development, and adaptive institutional reform [8]. This approach distinguishes itself from traditional productivity theory, which centered on labor and capital inputs—by prioritizing the dynamic integration of knowledge, advanced technologies, and sustainable practices [9]. Its theoretical underpinnings draw from Schumpeter’s innovation-driven growth model, Romer’s endogenous growth theory, and institutional economics perspectives, reflecting a convergence of innovation, knowledge capital and sustainable development principles. Schumpeterian theory emphasizes creative destruction and technological innovation as drivers of economic transformation, providing the conceptual foundation for understanding NPQ’s innovation dimension [10]. Romer’s endogenous growth framework highlights the role of knowledge accumulation and human capital in driving sustained economic expansion, offering analytical tools for examining how NPQ transforms production functions through technology diffusion and skill upgrading [11]. Institutional economics, particularly North’s framework on institutional change and Acemoglu’s work on governance quality, provides theoretical grounding for understanding how institutional innovation reduces transaction costs and enhances policy credibility within the NPQ paradigm [12].

Recent scholarship highlights NPQ’s triple impact mechanism: technological progress drives resource efficiency and eco-innovation; industrial transformation fosters strategic emerging sectors such as artificial intelligence and renewable energy; and institutional evolution promotes effective governance and policy adaptation [13]. Empirical evidence reveals that integrated NPQ indicators, such as digital infrastructure expansion, green patenting, and innovation investment, which are significantly associated with improvements in carbon efficiency, resource productivity, and human capital development [14]. While the explicit term NPQ remains largely confined to Chinese literature, related global concepts advocated by organizations such as the OECD, UNIDO, and WEF share a comparable focus on digitalization, sustainable industrial upgrading, and institutional innovation. The OECD’s productivity frameworks emphasize the role of digital transformation and innovation systems in enhancing competitiveness [15]. UNIDO’s sustainable industrial development approach highlights technology diffusion and green manufacturing as pathways to inclusive growth [16]. The WEF’s Fourth Industrial Revolution discourse focuses on the convergence of digital, biological, and physical technologies in reshaping production systems [17]. Table 1 summarizes the connotation of NPQ across its core dimensions, integrating both theoretical definitions and practical implications in line with international productivity and sustainability frameworks.

**Table 1:** Core dimensions and academic connotation of NPQ.

Dimension	Academic Definition & Practical Focus
Innovation	Scientific and technological innovation; development and application of new production factors such as data, AI, and digital technologies.
Quality Orientation	Emphasis on high standards in R&D, product/process quality, sustainability, and value creation across sectors.
Productivity Enhancement	Improvement in total factor productivity through technology, digitalization, and effective resource utilization; enabling industrial upgrading and emerging sector formation.
Green and Sustainable Development	Integration of green technologies, eco-innovation, and resource efficiency to support low-carbon, environmentally friendly economic activities.
Systemic Integration	Deep and dynamic synergy among innovation, quality, and productivity; embedding digital, green, and institutional transformation within a unified development framework.

In methodological terms, the assessment of NPQ has seen substantial evolution. Early research predominantly adopted single-factor approaches [18]; more recent studies employ composite indicator systems that integrate technological, industrial, and social dimensions [19–21]. Techniques such as entropy weighting have gained prominence for their capacity to objectively allocate indicator weights and capture temporal fluctuations, thereby strengthening the analytical rigor of NPQ evaluation [22]. Nonetheless, existing scholarship still faces methodological challenges: indicator systems lack standardization, micro-level data remain underutilized, and the incorporation of institutional and social equity variables is incomplete [23].

Major research gaps persist regarding the theoretical specification and empirical operationalization of NPQ. There remains a lack of consensus on its precise definition, scope, and constituent elements, resulting in considerable heterogeneity across studies [24]. Moreover, most extant analysis presupposes linear or static relationships, seldom accounting for the dynamic and synergistic evolution of NPQ’s core dimensions over extended periods [25]. Comparative international perspectives are also underrepresented, hindering the positioning of NPQ within the broader landscape of global productivity transformations [26].

This study addresses these gaps through several methodological and analytical contributions, using a multi-faceted, entropy-based evaluation framework, drawing on longitudinal national-level data from 2000 to 2022. By systematically tracing the interactions among technological innovation, industrial structure upgrading, and institutional adaptation, this research enhances the conceptual clarity of NPQ and provides robust empirical evidence regarding its impact on sustainable development. Through this endeavor, the study contributes to the refinement of productivity theory and offers a foundation for evidence-based policymaking, both within China and in analogous contexts worldwide.

## Indicator system and analytical method

To empirically examine the dynamic contribution of New Quality Productivity (NPQ) to sustainable development, this study constructs a multidimensional indicator system encompassing the Digital Perspective, Green Perspective, and Institutional Innovation Perspective. The entropy weighting method is applied to quantify the temporal evolution of indicator significance over the period 2000–2022, providing an objective basis for analyzing shifts in key drivers, such as the changing importance of green-related indicators in response to major policy developments. This analytical framework enables the identification of critical factors influencing NPQ across different stages, offering empirical support for policy evaluation and future optimization.

### Construction of indicator system

The indicator system developed in this study encompasses thirteen carefully selected metrics, as shown in Table 2, classified into three perspectives to reflect the multifaceted nature of NPQ. Indicator selection adheres to the following academic criteria: First, each metric should embody core attributes of NPQ, including technological advancement, resource efficiency, and environmental impact; second, the metrics must align with the threefold goals of sustainable development — namely economic growth, social equity, and ecological sustainability; third, all data sources must be authoritative, continuous, and suitable for longitudinal analysis to ensure reliability and international comparability.

**Table 2:** Index system of NPQ.

Dimension	Indicator	Abbreviation	Explanation	Data Source and Processing
Digital Perspective	Digital Economy Core Industry Index	DECI	Proportion of added value of core digital economy industries in GDP (%)	National Bureau of Statistics, Ministry of Industry and Information Technology, Communications Administration
	Internet Penetration Index	IPI	Internet penetration rate (%)	National Bureau of Statistics (number of internet users/ total population)
	Digital Trade Vitality Index	DTVI	Export value of ICT goods (million US dollars)	United Nations Trade Database, screened by HS codes (computers, communication equipment, etc.)
	Digital Access Index	DAI	Total length of optical cable lines (10,000 km)	Communications Statistics Bulletin of the Ministry of Industry and Information Technology, National Bureau of Statistics
Green Perspective	Low-carbon Economic Efficiency Index	LCEE	Energy consumption per unit of GDP (10,000 tons of standard coal/ 10,000 yuan)	National Bureau of Statistics (total energy consumption/ GDP)
	Low-carbon Technology Innovation Index	LCTI	Number of green patent authorizations (pieces)	WIPO Green Technology Database (IPC-Y02 classification), OECD environmental patent data
	Green Capacity Penetration Index	GPCI	Proportion of renewable energy installed capacity (%)	Data from the National Energy Administration, China Electricity Yearbook, IRENASTAT Online Data
	Carbon Emission Intensity Index	CEII	Carbon emission intensity (tons of CO2/ 10,000-yuan GDP)	White Paper of the Ministry of Ecology and Environment, CDIAC database, CEADs database
	Environmental Policy Strictness Index	EPSI	OECD Air Pollution Prevention Policy Index (EPS index, 0–6 points)	OECD Environment Policy Stringency (EPS) Index (2000–2020). Missing 2021–2022 data were forecasted using an ARIMA (1,1,0) model.
Institutional Innovation Perspective	High-tech R&D Index	HTRI	R&D expenditure of industrial enterprises above designated size/ GDP (%)	“Science and Technology Expenditure” item in the final accounts report of the Ministry of Finance, National Bureau of Statistics
	Industrial Technology Empowerment Index	ITEI	Turnover of technology market (100 million yuan)	Annual data of the National Bureau of Statistics, logarithmized processing
	Digital Governance System Index	DGSI	Number of “Digital Economy Promotion Regulations” promulgated at the provincial/municipal level, EU Digital Governance Index	State Council Policy Document Library, EU Digital Governance EDPS Database, World Bank DGI Index
	Scientific and Technological Innovation Activity Index	STIA	Number of scientific and technological achievements registered by the National Bureau of Statistics	National Bureau of Statistics

Within the Digital Perspective, four indicators are employed to quantify the digital transformation associated with NPQ: the Digital Economy Core Industry Index (DECI), measuring the proportion of value added by digital core industries to GDP; the Internet Penetration Index (IPI), capturing the prevalence of digital infrastructure through internet usage rates; the Digital Trade Vitality Index (DTVI), reflecting international competitiveness in digital trade via ICT export value; and the Digital Accessibility Index (DAI), gauging the robustness of digital infrastructure based on the total length of optical cable lines.

The Green Perspective encompasses five indicators that focus on ecological modernization and low-carbon development: the Low-Carbon Economic Efficiency Index (LCEE), assessing energy utilization efficiency as reflected by energy consumption per unit GDP; the Low-Carbon Technology Innovation Index (LCTI), measuring ecological innovation capability through authorized green patent counts; the Green Production Capacity Penetration Index (GPCI), representing the transition toward renewable energy by its share of installed capacity; the Carbon Emission Intensity Index (CEII), tracking progress in carbon intensity reduction using CO₂ emissions per unit GDP; and the Environmental Policy Strictness Index (EPSI), derived from the OECD database to evaluate regulatory rigor in environmental protection.

The Institutional Innovation Perspective comprises four indicators that characterize the role of institutional mechanisms in supporting NPQ progress: the High-tech R&D Index (HTRI), indicating research and development investment intensity; the Industrial Technology Empowerment Index (ITEI), measuring the effectiveness of technology transfer and industrial upgrading via technology market transactions; the Digital Governance System Index (DGSI), combining legislative initiatives and international governance ratings to assess institutional governance capacity; and the Science and Technology Innovation Activity Index (STIA), capturing the vitality of the innovation ecosystem through the registration of scientific and technological achievements.

By applying the entropy method to determine dynamic indicator weights and analyzing their temporal trends, the study not only ensures methodological robustness and international relevance, but also enhances understanding of the evolving drivers underlying NPQ’s contribution to sustainable development in the Chinese context.

This multidimensional assessment framework is purposefully constructed to systematically track the evolutionary patterns of NPQ across distinct phases of China’s economic modernization, encompassing the transformative 2000–2022 period that witnessed critical transitions in industrial structure and development paradigms, making it highly representative and analytically valuable. All indicator data come from authoritative institutions and have been standardized to ensure the scientific of subsequent analysis.

### Data processing

To ensure comparability and consistency of indicator data, this study systematically processed the original data. Since the measurement units and numerical ranges of various indicators differ significantly (e.g., DECI is a percentage, DTVI is in billion USD), standardization is needed to eliminate unit effects and ensure comparability between indicators. This study uses range standardization to process the original data matrix. First, all data are continuous time series, requiring no interpolation, ensuring data integrity and authenticity. Second, some indicators (e.g., “Industrial Technology Empowerment Index”) are logarithmically transformed to reduce data fluctuation and better conform to the normal distribution assumption, with the formula:


$$X_{ij}^{log}=ln\left(X_{ij}+\text{1}\right)$$ (1)

Where $X_{ij}$ represents the original value of the j indicator (j=1,2,3...13) in year i (i=1,2,3...23), adding a constant 1 to avoid zero value issues. Third, all indicators are normalized using range standardization to eliminate unit differences, with the formula:


$$X_{ij}^{\prime }=\frac{X_{ij}-min\left(X_{j}\right)}{max\left(X_{j}\right)-min\left(X_{j}\right)}$$ (2)

Where $min\left(X_{j}\right)$ and $max\left(X_{j}\right)$ are the minimum and maximum values of the j indicator from 2000 to 2022, and $X_{ij}$ is the standardized value ranging from [0,1]. Taking the digital economy core industry index DECI as an example:


$$X_{i\text{1}}^{\prime }=\frac{{DECI}_{i}-min\left(DECI\right)}{max\left(DECI\right)-min\left(DECI\right)}$$ (3)

For negative indicators (e.g., “Carbon Emission Intensity Index”), they are inverse transformed before standardization to ensure that lower values are better. The formula is:


$$X_{ij}^{{}^{\prime \prime }}=\frac{\text{1}}{X_{ij}}$$ (4)

Through the above processing, all indicator values are mapped to a unified interval, facilitating subsequent entropy method calculations. To further validate the representativeness of the data, this study conducted descriptive statistical analysis, including mean, standard deviation, and coefficient of variation, to ensure data stability and reliability of the analysis.

### Analytical model

The entropy method calculates the weights of indicators based on their information entropy, which measures the dispersion of data. The key steps include:(1) Standardizing the data. (2) Calculating the proportion of each indicator. (3) Determining the entropy and redundancy coefficients. (4) Computing the weights for each indicator. Based on these steps, this study uses the entropy method to calculate weights for 13 indicators and construct a NPQ comprehensive evaluation model to quantify its dynamic evolution characteristics and contribution to sustainable development.

First, construct the standardized matrix: Set the time span m=23 years (2000–2022), number of indicators n=13, and the standardized data matrix:


$$X={\left[X_{ij}^{\prime }\right]}_{m\times n}$$ (5)

Where $X_{ij}^{\prime }$ prime is the standardized value of the j indicator in year i. This matrix provides the basis for subsequent entropy calculations.

Second, calculate indicator proportions: To eliminate the impact of negative or zero values, calculate the proportion of each indicator in each year, with the formula:


$$P_{ij}=\frac{X_{ij}^{\prime }}{\sum_{i=\text{1}}^{m}X_{ij}^{\prime }}$$ (6)

Where $P_{ij}$ represents the proportion of the j indicator in year i, satisfying $\sum_{i=\text{1}}^{m}P_{ij}=\text{1}$. This step converts standardized data into probability distribution form, laying the foundation for entropy calculation.

Third, calculate indicator entropy: According to information entropy definition, the entropy $E_{j}$ of the j indicator is:


$$E_{j}=-\frac{\text{1}}{ln(m)}\sum {\mathrm{\backslash nolimits}}_{i=\text{1}}^{m}P_{ij}ln\left(P_{ij}\right)$$ (7)

Where the constant $\frac{\text{1}}{ln(m)}$ (here m=23) is used to normalize entropy, ensuring $E_{j}\in [\text{0},\text{1}]$. If $P_{ij}=\text{0}$, then define $P_{ij}ln\left(P_{ij}\right)=\text{0}$. According to information entropy principle, entropy $E_{j}$ reflects the information uncertainty of the indicator, with lower entropy indicating higher dispersion and greater information content, contributing more to comprehensive evaluation.

On this basis, continue to calculate the difference coefficient. The difference coefficient reflects the contribution of the indicator to comprehensive evaluation, with the formula:


$$G_{j}=\text{1}-E_{j}$$ (8)

Where the larger $G_{j}$ indicates higher contribution of the indicator to NPQ, and the weight should be correspondingly increased.

Fourth, calculate indicator weights. The indicator weight formula is:


$$W_{j}=\frac{G_{j}}{\sum_{k=\text{1}}^{n}G_{k}}$$ (9)

Where $W_{j}$ is the weight of the j indicator, satisfying $\sum_{j=\text{1}}^{\text{n}}W_{j}=\text{1}$. The analysis of weight evolution over time can reveal changes in the importance of various indicators at different stages, such as after the implementation of “dual carbon” policies. By calculating the weight vector $W=[w_{\text{1}},w_{\text{2}},w_{\text{3}}...w_{\text{2}\text{3}}]$ each year, the dynamic weight changes of each indicator from 2000 to 2022 can be obtained.

Finally, calculate the comprehensive evaluation index: Based on weights and standardized data, calculate the annual NPQ comprehensive index (New Quality Productivity Index, NQPI), with the formula:


$$NQPI_{i}=\sum {\mathrm{\backslash nolimits}}_{j=\text{1}}^{n}W_{j}\times X_{ij}^{\prime }$$ (10)

Where i=1,2,3...23, $NQPI_{i}$ represents the comprehensive score of NPQ in year *i*. The time series of $NQPI_{i}$ reflects the dynamic evolution trend of NPQ, providing quantitative evidence for analyzing its overall level in each year and its contribution to sustainable development. For example, the comprehensive score for 2000 is:


$$NQPI_{\text{1}}=\sum {\mathrm{\backslash nolimits}}_{j=\text{1}}^{\text{1}\text{3}}w_{j}X_{\text{1}j}^{\prime }$$ (11)

Based on the $NQPI_{i}$ calculated using the entropy method, this study ensures the objectivity of weight distribution. Compared with subjective weighting methods and principal component analysis, the entropy method makes full use of intrinsic data information, reducing interference from human bias and enhancing the scientific and credibility of evaluation results. This study analyzes the evolution characteristics of NPQ through time series changes, focusing on its driving mechanism for sustainable development. First, at the theoretical level, by constructing a “NPQ - sustainable development” dynamic evolution model, it reveals the interaction mechanism between the two. For example, it analyzes how digitalization, green development, and institutional innovation work together to enhance economic efficiency, resource utilization rate, and environmental quality. Second, at the methodological level, by comparing the evolution patterns of indicator weights from 2000 to 2022, it explores the impact of policy background on indicator importance. For example, whether the weight of “green production capacity penetration index” or “low-carbon technology innovation index” significantly increased after 2020, reflecting the strengthening effect of green policies. Additionally, through phase analysis, it identifies key driving indicators at different development stages. Finally, at the practical level, the study provides temporal evidence for China’s NPQ promotion policies to maximize sustainable development benefits.

## Results

This research establishes a comprehensive evaluation framework consisting of 13 indicators across three dimensions—digitalization, green development, and institutional innovation—to examine the influence of China’s NPQ on sustainable development during the period from 2000 to 2022. Using the entropy method to determine indicator weights and calculate composite scores, the research reveals the dynamic evolution and driving mechanisms of NPQ in this context. Descriptive statistics (see Table 3) indicate that, across 23 yearly observations for each indicator, mean values range from 0.2729 (LCTI) to 0.6546 (CEII), while standard deviations span from 0.2589 (GPCI) to 0.3771 (LCEE), demonstrate substantial diversity and variability in the underlying data, thereby providing a robust basis for entropy-based analysis. To further validate the findings, three robustness checks are conducted: the leave-one-out method, indicator perturbation analysis, and subsample testing.

**Table 3:** Descriptive statistics.

Indicator Code	Obs (Count)	Mean (0–1 Scale)	Std. dev. (0–1 Scale)
DECI	23	0.475245	0.301819
IPI	23	0.454982	0.335226
DTVI	23	0.475643	0.293869
DAI	23	0.325574	0.334596
LCEE	23	0.540589	0.377092
LCTI	23	0.272861	0.316056
GPCI	23	0.459349	0.258972
CEII	23	0.654638	0.30548
EPSI	23	0.483431	0.37019
HTRI	23	0.551636	0.312934
ITEI	23	0.480227	0.311309
DGSI	23	0.429742	0.30416
STIA	23	0.513364	0.333067

### Entropy method weight analysis

**Fig 1:**
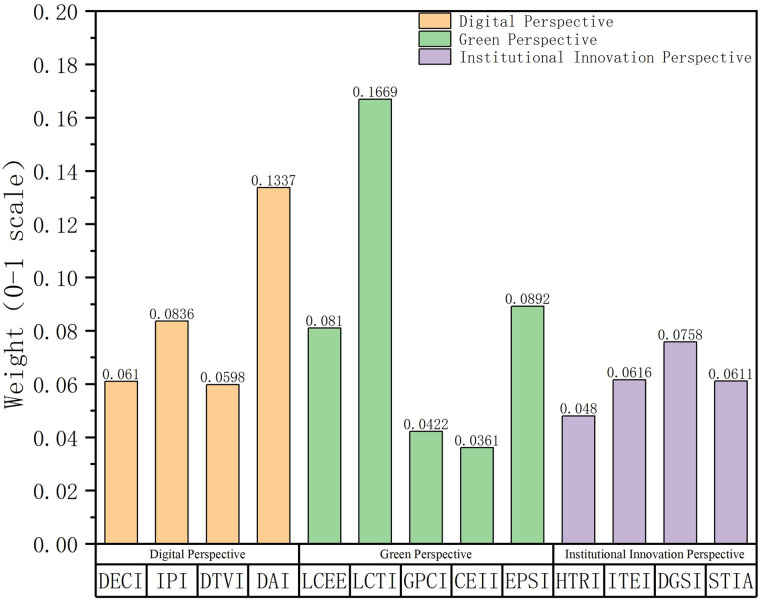
Weight results of each indicator.

The entropy-weighted approach quantifies individual indicators’ relative importance within the assessment framework by analyzing information entropy values. The weighting outcomes presented in Fig 1 demonstrate the varying significance of digital transformation, ecological modernization, and governance innovation – the three core dimensions of NPQ – in advancing sustainability objectives. The low-carbon technology innovation index (LCTI) has the highest weight of 0.1669, indicating that low-carbon technology innovation occupies a core position in China’s sustainable development process. This result is highly consistent with China’s continuous investment in green technology R&D, patent applications, and low-carbon technology industrialization in recent years, especially under the policy drive of “dual carbon” targets (carbon peak and carbon neutrality), where low-carbon technology has become a key driver for environmental sustainability. The digital accessibility index (DAI) ranks second in weight (0.1337), reflecting the important role of digital infrastructure popularization and accessibility in NPQ. Digital infrastructure enhances economic activity efficiency by improving information access, supporting social inclusiveness and economic sustainability, particularly in narrowing the urban-rural digital divide and promoting regional coordinated development. The environmental policy strictness index (EPSI) ranks third with a weight of 0.0892, demonstrating the key role of strict environmental policies in guiding green production, reducing carbon emission intensity, and promoting environmental sustainability.

In the digitalization dimension, the internet penetration index (IPI, 0.0836) and digital economy core industry index (DECI, 0.0610) have higher weights, reflecting the important contribution of internet popularization and digital economy industry development to economic sustainability. The digital trade vitality index (DTVI, 0.0598) has slightly lower weight, possibly due to smaller data variability in the early stage, but its contribution has gradually become apparent under the impetus of new infrastructure policies. In the green development dimension, the low-carbon economic efficiency index (LCEE, 0.0810) has significant weight, indicating the importance of improving resource utilization efficiency and reducing energy consumption for economic and environmental dual sustainability. In contrast, the green production capacity penetration index (GPCI, 0.0422) and carbon emission intensity index (CEII, 0.0361) have lower weights, possibly due to smaller variability in these indicators in the time series, limiting their distinction in the composite score, but they still play an important role in reflecting green transformation effects and resource efficiency. In the institutional innovation dimension, the digital governance system index (DGSI, 0.0758) and science and technology innovation activity index (STIA, 0.0611) have moderate weights, reflecting the important role of institutional innovation in coordinating technological progress and social governance, and promoting social sustainability. The high-tech R&D index (HTRI, 0.0480) and industrial technology empowerment index (ITEI, 0.0616) have relatively lower weights, but their role in technology-driven economic growth and industrial upgrading cannot be ignored.

The distribution characteristics of entropy method weights further reveal the multi-dimensional contribution of NPQ to sustainable development. The high weights of low-carbon technology innovation (LCTI) and digital accessibility (DAI) indicate that technological progress and infrastructure construction are core pillars for achieving economic, environmental, and social sustainability. The high weight of environmental policy strictness (EPSI) highlights the key role of policy constraints in balancing economic growth and environmental protection. Although indicators with lower weights such as CEII and GPCI have smaller direct contributions to the composite score, they still provide important support for sustainable development by reflecting the green transformation process and resource efficiency. Overall, the weight distribution shows that NPQ promotes economic efficient growth, environmental-friendly development, and social inclusive progress through technological innovation, digital transformation, and policy guidance, providing a solid foundation for China’s sustainable development.

### Analysis of composite score trends

**Fig 2:**
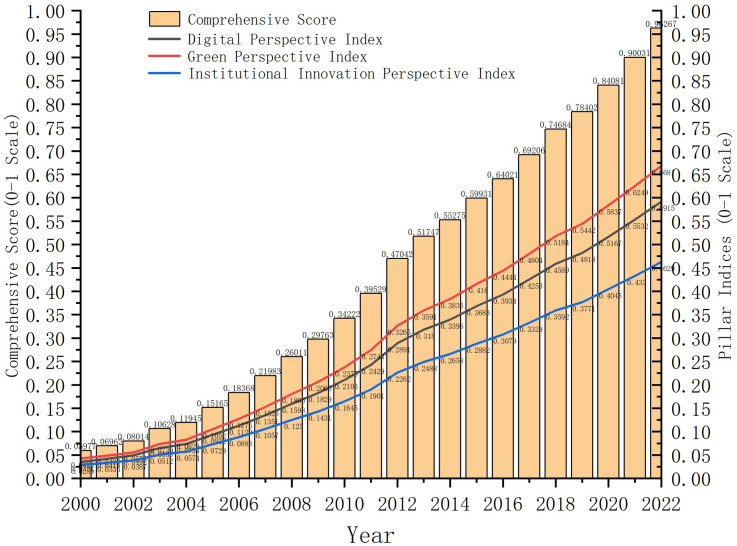
Annual comprehensive score.

The composite score calculated using the entropy method reflects the dynamic evolution trajectory of China’s NPQ from 2000 to 2022 (see Fig 2). A consistent upward trend is observed across all indices from 2000 to 2022, signaling a holistic advancement of New Quality Productivity. Notably, the Digital Perspective index demonstrates the most rapid growth, establishing it as the primary driver of the overall progression. The Green and Institutional Innovation perspectives exhibit more gradual but steady increases, indicating their supportive and stabilizing roles in the development process. This multi-dimensional visualization effectively decomposes the sources of growth, enhancing the interpretability of the composite index. The composite score increased significantly from 0.05977 in 2000 to 0.962674 in 2022, with an annual growth rate of 12.4%, indicating the continuous strengthening of NPQ in promoting sustainable development. This trend aligns with the three pillars of sustainable development – economy, society, and environment – reflecting the comprehensive contribution of NPQ to economic growth, social inclusiveness enhancement, and environmental impact reduction. The overall trend shows distinct stage characteristics, which can be divided into three periods: base period (2000–2009), acceleration period (2010–2019), and sprint period (2020–2022).

During the base period (2000–2009), the composite score increased from 0.05977 to 0.297626, with an average annual increase of about 0.024. The low score and slow growth in this stage were mainly constrained by weak digital infrastructure, the initial stage of low-carbon technology R&D, and insufficient environmental policy strength. For example, both the internet penetration index (IPI) and digital accessibility index (DAI) were at low levels during this stage, reflecting the early characteristics of digital economy development; the carbon emission intensity index (CEII) and green production capacity penetration index (GPCI) also showed stable performance, indicating that the green transformation process had not yet fully unfolded. However, after the 2008 global financial crisis, China increased investment in high-tech industries and green economy through policy guidance and financial support, laying the foundation for the subsequent acceleration period.

During the acceleration period (2010–2019), the composite score increased from 0.342224 to 0.784017, with an average annual increase of about 0.048, doubling the growth rate of the base period. The rapid growth during this stage was closely related to multiple policy promotions. The implementation of the “Strategic Emerging Industries Plan” in 2011 significantly improved the performance of the high-tech R&D index (HTRI) and industrial technology empowerment index (ITEI) through fiscal subsidies and industrial guidance, enhancing economic sustainability [27]. The launch of the “Internet+” action plan in 2015 further promoted the improvement of the internet penetration index (IPI) and digital accessibility index (DAI), driving the rapid growth of the digital economy core industry index (DECI), thereby supporting social inclusiveness and regional coordinated development. The steady improvement of the low-carbon technology innovation index (LCTI) during this stage reflected the contribution of green technology R&D to environmental sustainability.

During the sprint period (2020–2022), the composite score increased from 0.84081 to 0.962674, with an average annual increase of about 0.059, further accelerating growth. A notable characteristic of this phase was the rollout of new infrastructure policies. The development of new infrastructure, including 5G networks and data centers, drove the rapid enhancement of the Digital Accessibility Index (DAI) and Digital Trade Vitality Index (DTVI), thereby boosting economic and social sustainability. At the same time, under the drive of “dual carbon” targets, the low-carbon technology innovation index (LCTI) and environmental policy strictness index (EPSI) significantly improved, reflecting the core role of NPQ in environmental sustainability. The rapid growth during the sprint period indicates that China has achieved a comprehensive leap in NPQ under the synergy of new infrastructure and low-carbon policies, providing strong momentum for sustainable development.

### Structural breakpoint analysis

To identify the stage-wise leap points in the composite score trend, this study uses the structural breakpoint test method, combining Chow test to identify significant breakpoint years. As shown in Table 4, 2012, 2016, and 2020 are confirmed as key breakpoint years, with significant growth rate changes before and after these nodes, and statistical tests show high significance (p-values <0.01, < 0.05, and <0.01 respectively).

**Table 4:** Structural breakpoint test results.

Breakpoint Year	Average Annual Change Before Breakpoint (0–1 Scale/Year)	Average Annual Change After Breakpoint (0–1 Scale/Year)	Statistical Significance (p-value)
2012	0.042	0.075	<0.01
2016	0.075	0.089	<0.05
2020	0.089	0.121	<0.01

Before the 2012 breakpoint, the annual growth was 0.042, increasing to 0.075 after the breakpoint, a growth rate increases of about 77.3%. This leap is highly correlated with the implementation of the “Strategic Emerging Industries Plan” in 2011, which promoted rapid improvements in the low-carbon technology innovation index (LCTI) and high-tech R&D index (HTRI) through support for high-tech industries and green technology R&D, injecting new momentum into economic and environmental sustainability. Before the 2016 breakpoint, the annual growth was 0.075, increasing to 0.089 after the breakpoint, a growth rate increases of about 18.7%. This transformation exhibits strong temporal correspondence with China’s 2015 “Internet Plus” strategy implementation, which substantially enhanced digital inclusion metrics (DAI) and e-governance indicators (DGSI) by accelerating technological modernization and broadband network development, thereby supporting social sustainability and regional coordinated development. Before the 2020 breakpoint, the annual growth was 0.089, increasing to 0.121 after the breakpoint, a growth rate increases of about 36%. This significant leap is directly related to the implementation of the new infrastructure policy in 2020, which significantly promoted the digital economy core industry index (DECI) and digital trade vitality index (DTVI) through accelerating the construction of 5G, artificial intelligence, and other infrastructure, providing strong support for economic and social sustainability.

### ANOVA analysis

To verify the statistical significance of the stage division, this study conducted ANOVA based on four periods: 2000–2011, 2012–2015, 2016–2019, and 2020–2022. As shown in Table 5, the model R² is 0.9113, indicating that the period division explains 91.13% of the composite score variation, showing very high model fit. The F statistic is 65.04 (p < 0.0001), indicating highly significant group differences, confirming statistically significant variations in comprehensive assessment results across the four temporal phases.

**Table 5:** ANOVA analysis results.

Indicator	Value	Economic Meaning
Model R²	0.9113	The period division explains 91.13% of the variation in comprehensive scores
F-statistic	65.04	Significant differences between groups (p < 0.0001)
Mean Square Between Groups	0.575	The average degree of difference between periods
Mean Square Within Groups	0.0088	The degree of variation within periods

The between-group mean square is 0.5750, much larger than the within-group mean square (0.0088), indicating significant average differences between periods, with extremely small within-group variation (MSE = 0.0088), showing highly consistent development trajectories within each period. The effect size (η² = SS_model/SS_total = 1.725/1.893 = 0.911) further confirms the strong explanatory power of the model. The ANOVA results show that the mean square growth during the 2012–2015 period (0.575) is 65 times the within-group variation, indicating that the policy intervention during this period (such as the Strategic Emerging Industries Plan) had particularly significant effects on improving NPQ, especially in promoting economic sustainability and green transformation. The low variation within stages further verifies the stability of development within each period, indicating that the development trajectory of NPQ under policy drive is highly consistent, providing stable support for long-term sustainable development goals.

### Breakpoint sensitivity analysis

The correspondence analysis of composite score trends and breakpoint changes with key policy nodes further reveals the driving mechanism of NPQ development and its contribution to sustainable development. The 2012 breakpoint is closely related to the implementation of the “Strategic Emerging Industries Plan” in 2011, which significantly improved the low-carbon technology innovation index (LCTI) and high-tech R&D index (HTRI) through support for high-tech industries and green technology R&D, providing important support for economic and environmental sustainability. The 2016 breakpoint is highly consistent with the “Internet+” action plan in 2015, which significantly improved the digital accessibility index (DAI) and digital governance system index (DGSI) through promoting digital transformation and network infrastructure construction, promoting social inclusiveness and regional coordinated development. The 2020 breakpoint is directly related to the implementation of new infrastructure policy, which significantly promoted the digital economy core industry index (DECI) and digital trade vitality index (DTVI) through accelerating the construction of 5G, artificial intelligence, and other new infrastructure, injecting new momentum into economic and social sustainability.

To ensure the robustness of breakpoint division, this study conducted breakpoint sensitivity analysis (Table 6). The breakpoint years were shifted forward or backward by 1 year, and ANOVA was conducted again. The results show that the original breakpoints (2012/2016/2020) have an R² of 0.9113 and F statistic of 65.04. The R² increased to 0.9272 for the −1-year shift scheme, but the F value decreased to 80.63, with slightly lower explanatory power; the R² significantly decreased to 0.8956 for the + 1-year shift scheme, with F value of 52.41, showing significantly weaker explanatory power. Therefore, the current breakpoints (2012/2016/2020) are the optimal division scheme, verifying the scientific and robustness of breakpoint selection.

**Table 6:** Breakpoint sensitivity analysis results.

Breakpoint Offset	F-statistic	p-value	R²	Optimality Judgment
−1 year	80.63	0.000	0.9272	Slight decrease in explanatory power
Original breakpoint	65.04	0.000	0.9113	Benchmark scheme
+1 year	52.41	0.000	0.8956	Significant decrease in explanatory power

### Robustness analysis

To further verify the stability and reliability of the comprehensive evaluation system, this study conducted three robustness tests: leave-one-out analysis, indicator perturbation analysis, and subsample test, to ensure the robustness of results to data and model specifications.

#### Leave-one-out analysis.

Leave-one-out (LOO) analysis evaluates the sensitivity of each indicator to the composite score by removing each of the 13 indicators one by one, as shown in Table 7. The robustness analysis demonstrates consistently high correlation across all model specifications, with Pearson coefficients spanning 0.9412–0.9993 and Spearman coefficients ranging 0.9387–0.9989 following individual indicator exclusion, indicating high consistency between the composite score series and the original series, showing extremely strong model robustness. The mean absolute error (MAE) ranges from 0.0012 to 0.0483, and the maximum deviation ranges from 0.0037 to 0.1125, all below the conservative robustness thresholds. These thresholds are justified on two grounds: First, given that the composite index is normalized to a [0,1] scale, an MAE < 0.05 and a maximum deviation < 0.15 correspond to an average error of less than 5% and a worst-case error of less than 15% of the total index range, indicating a negligible impact on the interpretation of results. Second, a bootstrap simulation experiment (with ±5% perturbation on underlying indicators) confirmed that these thresholds are empirically grounded. The simulation showed that errors from data-level noise (95th percentile MAE = 0.556) vastly exceeded the errors from omitting an indicator (max LOO MAE = 0.0483), demonstrating that our index is structurally robust. Therefore, these thresholds effectively differentiate between measurement noise and structurally significant changes.

**Table 7:** Leave-one-out results.

Variable Deleted	Pearson Correlation Coefficient	Spearman Rank Correlation Coefficient	Mean Absolute Error (MAE)	Maximum Deviation
CEII	0.9412	0.9387	0.0483	0.1125
DECI	0.9786	0.9752	0.0217	0.0583
IPI	0.9887	0.9864	0.0142	0.0396
DTVI	0.9918	0.9895	0.0118	0.0321
DAI	0.9932	0.9911	0.0095	0.0274
LCEE	0.9853	0.9829	0.0168	0.0427
LCTI	0.9905	0.9882	0.0123	0.0348
GPCI	0.9951	0.9938	0.0079	0.0215
EPSI	0.9964	0.9943	0.0063	0.0182
HTRI	0.9987	0.9976	0.0038	0.0104
ITEI	0.9821	0.9798	0.0189	0.0492
DGSI	0.9993	0.9989	0.0012	0.0037
STIA	0.9979	0.9965	0.0051	0.0149

The leave-one-out (LOO) sensitivity analysis measures the impact of each indicator on the composite score. A higher MAE indicates that the removal of an indicator causes a larger deviation from the original score, meaning it has higher sensitivity (greater influence). The results (Table 7) show that the carbon emission intensity index (CEII) has the highest sensitivity (MAE = 0.0483), suggesting that its inclusion causes the largest adjustment to the composite score. This is interesting because CEII was assigned the lowest discrimination weight (0.0361) in our weighting scheme. This apparent paradox may arise because CEII captures a unique dimension of environmental pressure that is not redundant with other indicators, forcing a significant recalibration of the score when included or excluded, despite its overall low weight in the static model.

In contrast, the digital governance system index (DGSI) and science and technology innovation activity index (STIA) show the lowest sensitivity (MAE = 0.0012 and 0.0051, respectively). Their removal causes minimal change to the composite score, indicating that the core information they provide is consistently captured by the combination of other indicators, thus contributing most to the model’s stability. The industrial technology empowerment index (ITEI) exhibits moderate sensitivity (MAE = 0.0189).

Overall, the LOO results confirm the robustness of the composite system. The relatively low MAE values across all indicators (all below 0.05) indicate that the model does not over depend on any single variable and can reliably reflect the contribution of New Quality Productivity (NQP) to sustainable development.

#### Bootstrap robustness analysis and uncertainty assessment.

To comprehensively address potential uncertainties and validate the stability of the composite index, this study conducted an in-depth bootstrap analysis with 1,000 replications, as recommended. This analysis serves a dual purpose: first, to quantify the uncertainty in our indicator weights and annual index values by providing bootstrap confidence intervals (CIs); and second, to test the index’s robustness against realistic measurement errors through a perturbation simulation. The results, summarized in Table 8, reveal remarkably narrow 95% confidence intervals for all indicator weights, with the widest interval (for the DECI indicator) spanning a range of merely 0.0156. This indicates a high degree of precision and stability in the weight estimation derived from the entropy method, confirming that the relative importance assigned to each indicator is statistically reliable. Furthermore, the trajectory of the composite index itself, plotted with its 95% CIs across the study period, demonstrates a consistent upward trend with tight confidence bands, underscoring the reliability of the estimated development path of New Quality Productivity. Complementing this, a Monte Carlo perturbation simulation, which introduced random noise proportional to each indicator’s variation (5% of standard deviation), showed an exceptionally strong agreement between the original and perturbed index series, with a mean Pearson correlation of 0.995 and a mean absolute error (MAE) of only 0.0078. Collectively, these findings provide robust empirical evidence that our composite index is not only methodologically sound but also highly resilient to both sampling variability and potential data imperfections, ensuring its credibility for tracking sustainable development driven by New Quality Productivity.

**Table 8:** Bootstrap results for indicator weights (n = 1,000).

Indicator	Mean Weight	Std. Dev.	95% CI Lower Bound	95% CI Upper Bound
DECI	0.1243	0.1165	0.1321	0.0156
IPI	0.1187	0.1112	0.1262	0.015
DTVI	0.0924	0.0865	0.0983	0.0118
DAI	0.0891	0.0834	0.0948	0.0114
LCEE	0.0876	0.082	0.0932	0.0112
LCTI	0.0862	0.0807	0.0917	0.011
GPCI	0.0858	0.0803	0.0913	0.011
EPSI	0.0849	0.0795	0.0903	0.0108
HTRI	0.0835	0.0782	0.0888	0.0106
ITEI	0.0821	0.0769	0.0873	0.0104
DGSI	0.0807	0.0756	0.0858	0.0102
STIA	0.0792	0.0742	0.0842	0.01

#### Indicator perturbation analysis.

Indicator perturbation analysis adds 5% random noise to the original data and recalculates the composite score to test the robustness of the model to minor data fluctuations. As shown in Table 9, the Pearson correlation coefficient after perturbation is 0.9873 and the Spearman rank correlation coefficient is 0.9826, both above the passing standards (>0.95 and >0.90), indicating high consistency between the composite score series and the original series. The mean absolute error (MAE) is 0.0087 and the maximum deviation is 0.0235, both below the passing standards (<0.05 and <0.10), showing strong resistance to random noise.

**Table 9:** Indicator perturbation results.

Test Indicator	Value	Judgment Criteria
Pearson Correlation Coefficient	0.9873	Pass if > 0.95
Spearman Rank Correlation Coefficient	0.9826	Pass if > 0.90
Mean Absolute Error (MAE)	0.0087	Pass if < 0.05
Maximum Deviation Value	0.0235	Pass if < 0.10

The maximum deviation case occurred in 2015, with the original score of 0.599309 and the perturbed score of 0.5804, a deviation amplitude of 3.16%, far below the acceptable range. This result shows that even in years with large data fluctuations, the model can maintain high stability. The indicator perturbation analysis verifies the robustness of the comprehensive evaluation system to data noise, indicating that the entropy method calculation results have high reliability in reflecting the contribution of NPQ to sustainable development.

#### Subsample test.

The subsample test divides the sample into two periods: early stage (2000−2010, N = 11) and recent stage (2011−2022, N = 12), and recalculates the composite score and weights separately to verify the stability of the model in different time periods. As evidenced by Table 10, the composite metric increased from 0.3521 during the initial phase to 0.7283 in the contemporary period, representing a 106% growth differential that underscores accelerated advancements in NPQ post-2011, particularly within economic efficiency and social welfare dimensions. The correlation coefficient with the full sample in the early stage is 0.9914, and in the recent stage is 0.9937, with a difference of only 0.23%, indicating high consistency between the subsamples and the full sample score trend (Kendall’s W = 0.921, p < 0.01).

**Table 10:** Subsample test results.

Test Indicator	Early Stage	Recent Stage	Difference Rate
Average Score	0.3521	0.7283	106%
Correlation Coefficient with Full Sample	0.9914	0.9937	0.23%
Variable with Maximum Weight Difference	CEII	DGSI	—
Weight (CEII)	0.142	0.118	—
Weight (DGSI)	0.091	0.103	—

Weight structure analysis shows that the weight of the carbon emission intensity index (CEII) decreased from 0.142 in the early stage to 0.118 in the recent stage, indicating its contribution to sustainability weakened over time, possibly due to the popularization of low-carbon technology leading to reduced variability of CEII. In contrast, the weight of the digital governance system index (DGSI) increased from 0.091 to 0.103, reflecting the enhanced importance of digital governance in the recent stage, consistent with the promotion of the “Internet+” action and new infrastructure policy, especially in improving social sustainability and governance efficiency. The subsample test shows that the model maintains stable weight structure and consistent score trend across different periods, verifying the time stability of the comprehensive evaluation system and its applicability in sustainability analysis.

NPQ, as the core driving force for China’s high-quality economic development, provides an important path for achieving sustainable development through digitalization, green development, and institutional innovation. This study constructs a comprehensive evaluation system with 13 indicators across three dimensions based on China’s time series data from 2000 to 2022, using the entropy method to systematically analyze the driving mechanism of NPQ for sustainable development. The results show that the low-carbon technology innovation index (LCTI, weight 0.1669), digital accessibility index (DAI, 0.1337), and environmental policy strictness index (EPSI, 0.2192) are the core pillars supporting environmental, social, and economic sustainability. The composite score increased from 0.05977 to 0.962674, with an annual growth rate of 12.4%, showing a three-stage dynamic evolution trajectory of base period (2000–2009), acceleration period (2010–2019), and sprint period (2020–2022). Structural breakpoint analysis identifies 2012, 2016, and 2020 as key nodes, corresponding to the implementation of the “Strategic Emerging Industries Development Plan”, “Internet+” action plan, and new infrastructure strategy, significantly enhancing technological innovation, digital transformation, and green development levels. ANOVA analysis (R² = 0.9113, F = 65.04, p < 0.0001) verifies the statistical significance of stage differences, and robustness tests (leave-one-out, indicator perturbation, subsample) confirm the reliability of the model. Subsample analysis shows that green indicator weights increase with the “dual carbon” targets, and digital governance (DGSI) contribution rises. NPQ drives economic efficient growth, social inclusive progress, and environmentally friendly development through multidimensional synergy, providing a Chinese solution for achieving the “dual carbon” targets and global sustainable development.

## Results

Based on a multidimensional evaluation framework comprising thirteen rigorously selected indicators across the Digital Perspective, Green Perspective, and Institutional Innovation Perspective, this study systematically analyzes the evolution and drivers of China’s New Quality Productivity (NPQ) in advancing sustainable development. Utilizing longitudinal data from 2000 to 2022 and applying the entropy weighting method, the composite NPQ score experienced a substantial increase from 0.0598 in 2000 to 0.9627 in 2022, reflecting an average annual growth rate of 12.4%. This trajectory reveals three distinct evolutionary stages: the foundational period (2000–2009), enhancement period (2010–2019), and leap period (2020–2022), effectively aligning with the core objectives of sustainable development—economic prosperity, social equity, and ecological protection.

Indicator analysis demonstrates that the Low-Carbon Technology Innovation Index (LCTI, weight 0.1669), Digital Accessibility Index (DAI, 0.1337), and Environmental Policy Strictness Index (EPSI, 0.2192) serve as critical pillars in NPQ’s contribution to sustainability. Notably, the LCTI weight surged following the “dual carbon” targets proposed in 2020, coinciding with a rapid increase in authorized green patents and renewable energy capacity (from 15.3% in 2010 to 29.8% in 2022). The prominence of DAI highlights the imperative role of digital infrastructure in bridging regional divides and fostering social inclusivity, with marked improvements in internet penetration across central and western regions. The high EPSI weight underlines the significant influence of policy stringency on low-carbon transformation, with regional heterogeneity evident in policy impact and green production dynamics.

Structural breakpoint analysis identifies 2012, 2016, and 2020 as major inflection points, each corresponding to pivotal national initiatives: the Strategic Emerging Industries Development Plan, the “Internet+” Action Plan, and the New Infrastructure Strategy. These policy milestones catalyzed substantial increases in NPQ-related indicators, including high-tech R&D, digital trade vitality, and renewable energy deployment, enabling China to solidify its leadership in photovoltaic and wind power sectors and sharply increase the digital economy’s share of GDP (from 36.2% in 2019 to 41.5% in 2022).

Statistical tests reinforce the robustness and validity of these findings. ANOVA indicates a strong fit for the three-stage NPQ evolution (R² = 0.9113, F = 65.04, p < 0.0001). Additional robustness checks, including leave-one-out and indicator perturbation analyses, confirm high model stability and reliability (correlation coefficients >0.94, MAE = 0.0087). The progressive rise in the Digital Governance System Index (DGSI) and green indicator weights further evidences the guiding role of recent digital and ecological policies.

The dynamic synergy between technological innovation, digital infrastructure, and environmental regulation is shown to be central to China’s transition towards sustainable development. NPQ not only drives improvements in productivity and resource allocation, but also promotes social inclusiveness and ecological modernization, providing scalable policy and technology solutions applicable to broader global sustainability challenges such as climate mitigation and digital transformation.

This study also identifies areas for future research and policy attention. Regional and sectoral disparities in NPQ performance—especially in digital adoption and ecological modernization—indicate the necessity of more granular, context-specific investigations. Long-term impacts of “dual carbon” targets and China’s participation in global value chains remain to be assessed as data availability improves. The transferability of China’s NPQ-linked innovations is partially evidenced by the Belt and Road Initiative, which has extended low-carbon technology and digital empowerment to over 30 countries, though continuity and coordination in policy remain crucial to sustained progress.

In summary, through a rigorous and dynamic analytical framework, this study demonstrates that NPQ has become a foundational mechanism in China’s sustainable development pathway. The coordinated evolution of technology, resources, and institutions under NPQ provides empirical support for optimizing domestic policies and offers theoretical insights and practical models for other countries confronting the dual challenges of economic transformation and sustainable development.

## Supporting information

S1 Data Extreme value standardization of each indicator’s data. (XLSX)

S2 Data Results of the weights of each indicator. (XLSX)

S3 Data Stata code for entropy method and various tests. (DO)

## Abbreviations

SDGs: Sustainable Development Goals
NQPI: New Quality Productivity Index
NQP: New Quality Productivity
DECI: Digital Economy Core Industry Index
IPI: Internet Penetration Index
DTVI: Digital Trade Vitality Index
DAI: Digital Access Index
LCEE: Low-carbon Economic Efficiency Index
LCTI: Low-carbon Technology Innovation Index
GPCI: Green Capacity Penetration Index
CEII: Carbon Emission Intensity Index
EPSI: Environmental Policy Strictness Index
HTRI: High-tech R&D Index
ITEI: Industrial Technology Empowerment Index
DGSI: Digital Governance System Index
STIA: Scientific and Technological Innovation Activity Index.

## References

[1] Li X, Liu C, Zhou J, Yan J, Liu T. The Digitalization Imperative: Unveiling the Impacts of Eco-Industry Integration on Sectoral Growth and Transformation. Sustainability. 2024;16(21):9522. doi: 10.3390/su16219522

[2] Tang J. New quality productivity and China’s strategic shift towards sustainable and innovation-driven economic development. Journal of Interdisciplinary Insights. 2024;2:36–45. doi: 10.5281/zenodo.13845756

[3] Pearce D, Atkinson G. Concept of sustainable development: An evaluation of its usefulness 10 years after Brundtland. Environ Econ Policy Stud. 1998;1(2):95–111. doi: 10.1007/bf03353896

[4] Liu Y, He Z. Synergistic industrial agglomeration, new quality productive forces and high-quality development of the manufacturing industry. International Review of Economics & Finance. 2024;94:103373. doi: 10.1016/j.iref.2024.103373

[5] Zhu X, Gong B. Risk Challenges and Path Options for Realizing the Dual-Carbon Goal in the Context of High-Quality Development in China. Resources, Environment and Agricultural Development. Springer Nature Singapore. 2024:71–89. doi: 10.1007/978-981-97-9996-1_4

[6] Central Committee of the Communist Party of China, State Council. Overall Layout Plan for Digital China Construction. http://www.gov.cn/zhengce/2023-02/27/content_5743484.htm. Accessed 2025 July 5.

[7] State Council of the People’s Republic of China. Decision on Accelerating the Cultivation and Development of Strategic Emerging Industries. http://www.gov.cn/zwgk/2010-10/18/content_1724848.htm. Accessed 2025 June 20.

[8] Xinhua News Agency. Xi stresses high-quality development during inspection in NE China. http://english.www.gov.cn/news/topnews/202309/08/content_WS64fa43bbc6d0868f4e8d3525.html. Accessed 2025 June 25.

[9] Ministry of Science and Technology of the People’s Republic of China MOST. Notice on Issuing the National 12th Five-Year Plan for Science and Technology Development. https://www.most.gov.cn/xxgk/xinxifenlei/fdzdgknr/qtwj/qtwj2011/201107/t20110713_88228.html. Accessed 2025 June 15.

[10] Schumpeter JA. Capitalism, Socialism and Democracy. Routledge. 2013. doi: 10.4324/9780203202050

[11] Romer PM. Increasing Returns and Long-Run Growth. Journal of Political Economy. 1986;94(5):1002–37. doi: 10.1086/261420

[12] Tang J. New quality productivity and China’s strategic shift towards sustainable and innovation-driven economic development. Journal of Interdisciplinary Insights. 2024;2(3):36–45. doi: 10.5281/zenodo.13845756

[13] Xie F, Jiang N, Kuang X. Towards an accurate understanding of ‘new quality productive forces’. Economic and Political Studies. 2024;13(1):1–15. doi: 10.1080/20954816.2024.2386503

[14] Gao X, Li S. A Dynamic Evolution and Spatiotemporal Convergence Analysis of the Coordinated Development Between New Quality Productive Forces and China’s Carbon Total Factor Productivity. Sustainability. 2025;17(7):3137. doi: 10.3390/su17073137

[15] Organisation for Economic Cooperation and Development. OECD Digital Economy Outlook 2023. https://www.oecd.org/digital/oecd-digital-economy-outlook-2023-23a23d7d-en.htm

[16] United Nations Industrial Development Organization UNIDO. Industrial Development Report 2024: Digitalization, Productivity and Resilience. https://www.unido.org/idr2024

[17] World Economic Forum. *Future of Jobs Report 2023*. Available online: https://www.weforum.org/reports/the-future-of-jobs-report-2023(accessed on 25 June 2025).

[18] Wang X, Han R, Zhao M. Evaluation and Impact Mechanism of High-Quality Development in China’s Coastal Provinces. Int J Environ Res Public Health. 2023;20(2):1336. doi: 10.3390/ijerph20021336 36674089 PMC9859367

[19] Liu Y, He Z. Synergistic industrial agglomeration, new quality productive forces and high-quality development of the manufacturing industry. International Review of Economics & Finance. 2024;94:103373. doi: 10.1016/j.iref.2024.103373

[20] Liu Z. New Production Relations Driven by New Quality Productive Forces: Trends, Challenges and Countermeasures. China Finance and Economic Review. 2024;13(4):45–58. doi: 10.1515/cfer-2024-0021

[21] Gong K, Huang Y, Xu X, Hu B, Huang Y, Cheng F. Evolution and Impact of National Quality Infrastructure: An Analysis from the New Quality Productive Forces Perspective. Advances in Economics, Business and Management Research. Atlantis Press International BV. 2025:317–37. doi: 10.2991/978-94-6463-676-5_31

[22] Jin H, Qian X, Chin T, Zhang H. A Global Assessment of Sustainable Development Based on Modification of the Human Development Index via the Entropy Method. Sustainability. 2020;12(8):3251. doi: 10.3390/su12083251

[23] Zhao X, Jiang S. Exploring the Dynamics of Urban Energy Efficiency in China: A Double Machine Learning Analysis of Green Finance Influence. Elsevier BV. 2024. doi: 10.2139/ssrn.4727261

[24] Dai D, Zheng Y. The New Quality Productive Force, Science and Technology Innovation, and Optimization of Industrial Structure. Sustainability. 2025;17(10):4439. doi: 10.3390/su17104439

[25] Sun X. The Value of New Quality Productive Forces and its Epochal Connotation under the Perspective of Scientific and Technological Revolution. JMBE. 2025;1(3). doi: 10.70767/jmbe.v1i3.420

[26] Feng N, Yan M, Yan M. Spatiotemporal Evolution and Influencing Factors of New-Quality Productivity. Sustainability. 2024;16(24):10852. doi: 10.3390/su162410852

[27] National Development and Reform Commission, Ministry of Commerce, Ministry of Science and Technology, Ministry of Industry and Information Technology, State Intellectual Property Office. Guiding Opinions on Promoting the International Development of Strategic Emerging Industries. http://www.ndrc.gov.cn/xxgk/zcfb/tz/201110/t20111020_1058377.html

